# CRISPR‐Cas9 screen reveals that inhibition of enhancer of zeste homolog 2 sensitizes malignant T cells to dimethyl‐fumarate‐induced cell death

**DOI:** 10.1111/febs.70208

**Published:** 2025-08-01

**Authors:** Jan P. Teubner, Deniz Tümen, Arne Kandulski, Philipp Heumann, Patricia Mester, Elisabeth Aschenbrenner, Kirstin Pollinger, Manuela Gunckel, Barbara Volz, Tobias Hein, Paul L. Beltzig, Luisa Tengler, Florian Voll, Marina Kreutz, Claudia Kunst, Jan P. Nicolay, Martina Müller, Karsten Gülow

**Affiliations:** ^1^ Department of Internal Medicine I, Gastroenterology, Hepatology, Endocrinology, Rheumatology, and Infectious Diseases University Hospital Regensburg Germany; ^2^ Department of Dermatology, Venereology and Allergology University Medical Center Mannheim/University of Heidelberg Mannheim Germany; ^3^ Department of Internal Medicine III, Hematology and Oncology University Hospital Regensburg Germany

**Keywords:** dimethyl fumarate (DMF), EZH2, histone methylation, lymphoma, Polycomb Repressive Complex 2 (PRC2)

## Abstract

Constitutive activation of the nuclear factor kappa‐light‐chain‐enhancer of activated B cells (NF‐κB) pathway is a hallmark of many lymphocyte‐associated cancers, including cutaneous T‐cell lymphoma (CTCL) and its leukemic variant, the Sézary syndrome. Dimethyl fumarate (DMF) has been identified as a promising NF‐κB‐targeted therapy and has shown positive outcomes in a phase II clinical trial involving patients with Sézary syndrome. However, limited responsiveness remains a significant challenge. Through a genome‐wide clustered regularly interspaced short palindromic repeats (CRISPR)‐Cas9 screen, we identified enhancer of zeste homolog 2 (EZH2; also known as histone‐lysine N‐methyltransferase) as a critical target for enhancing DMF‐induced cell death. EZH2, the catalytic subunit of Polycomb Repressive Complex 2 (PRC2), is responsible for the methylation of histone H3 (H3K27). Combining DMF with the US Food and Drug Administration (FDA)‐approved EZH2 inhibitor tazemetostat significantly increases cell death in patient‐derived CTCL cells, offering a promising strategy to improve therapeutic outcomes and overcome limited responsiveness to DMF.

## Graphical abstract

A genome‐wide CRISPR‐Cas9 screen identified H3K27 methylation as a key regulator of T‐cell lymphoma sensitivity to dimethyl fumarate (DMF). Specifically, inhibition of enhancer of zeste homolog 2 (EZH2) enhanced DMF‐induced cell death in malignant T cells. These results provide functional evidence for the role of EZH2 in modulating drug sensitivity and support further investigation of epigenetic modulation in the context of DMF treatment.

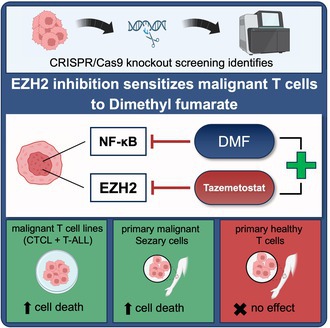

**Abbreviations**
CTCL: cutaneous T cell lymphoma
DMF: dimethyl fumarate
EZH2: Enhancer of Zeste Homolog 2
NF‐κB: nuclear factor kappa‐light‐chain‐enhancer of activated B cells
PRC2: Polycomb Repressive Complex 2
T‐ALL: T‐cell acute lymphoblastic leukemia

## Introduction

In certain T‐cell‐associated cancers, such as T‐cell acute lymphoblastic leukemia (T‐ALL), cutaneous T‐cell lymphoma (CTCL), and particularly its highly aggressive leukemic variant, the Sézary syndrome, constitutive NF‐κB activation is observed, which enables cells to evade apoptosis and drives uncontrolled proliferation [1, 2, 3]. Thus, targeting the NF‐κB pathway offers a promising strategy to enhance therapy and improve outcomes for patients with T‐ALL, CTCL, and Sézary syndrome, an aggressive leukemic variant of CTCL [4, 5, 6, 7].

Dimethyl fumarate (DMF), an FDA‐approved drug for the treatment of multiple sclerosis, shows promise as an NF‐κB inhibitor [6, 8]. DMF effectively suppresses NF‐κB in various malignant T‐cell populations [4, 5, 6, 9]. Our studies showed that in T‐ALL and CTCL cell lines, DMF inhibits NF‐κB by inactivating Thioredoxin‐1, leading to ripoptosome formation and cell death. Notably, both cell lines exhibited identical molecular mechanisms for DMF‐induced cell death, making T‐ALL cell lines an ideal model to study DMF‐affected signaling pathways. Furthermore, we confirmed in CD4+ T cells isolated from Sézary syndrome patients that DMF induces ripoptosome formation and cell death through NF‐κB inhibition [5, 6, 10]. In our recent multicenter phase II study (EudraCT number 2014‐000924‐11/NCT number NCT02546440), we show significant efficacy of DMF treatment in patients with CTCL. This led to a reduction in tumor burden and skin lesions [11]. These findings support DMF as a promising therapy for NF‐κB‐dependent T‐cell malignancies. Targeted therapies like DMF, with their low toxicity and minimal off‐target effects, are becoming essential in modern cancer treatment and may soon replace traditional chemotherapies in treatment guidelines [12, 13, 14]. A key limitation of targeted therapies is reduced treatment efficacy, often due to changes in the microenvironment and activation of bypass pathways [15, 16, 17]. To improve therapeutic outcomes, it is necessary to enhance drug efficacy by developing rational combination therapies. Modulating drug‐sensitizing genes is a promising approach, amplifying the impact of targeted therapies on malignant cells while maintaining tumor specificity. This facilitates low‐dose treatment, reducing toxicity to healthy cells while optimizing cancer cell elimination [18, 19]. To enhance DMF efficacy and improve responses in tumors with limited sensitivity, it is essential to identify strategies that increase or restore susceptibility to DMF treatment.

CRISPR‐based whole‐genome screens have significantly advanced our understanding of gene function, complex biological processes, and the molecular basis of diseases, therapeutic resistance, and responses to immunotherapy [20, 21, 22]. Compared to RNAi or cDNA library screens, CRISPR/Cas9‐based screens exhibit superior genetic editing with fewer off‐target effects, and increased versatility for large‐scale applications [23, 24, 25]. Recently, the CRISPR/Cas9‐based screens reliably identified drug sensitizer and resistance‐associated genes [26]. In addition, CRISPR/Cas9 screenings reveal cancer response mechanisms to drug treatment, offering insights into drug metabolism and gene pathway modulation [27].

We conducted a genome‐wide CRISPR/Cas9 knockout screen in a T‐ALL cell line to explore mechanisms of DMF‐induced cell death and to identify sensitizer genes. EZH2 and PRC2 were identified as key players, with efficacy confirmed in T‐ALL, CTCL, and Sézary patient CD4+ T cells. These findings highlight targeting PRC2 as a promising strategy for sensitizing NF‐κB‐dependent cancers to therapy, paving the way for future research and interventions.

## Results

### CRISPR/Cas9 knockout screening identifies EZH2 as a sensitizer for DMF‐induced cell death in T‐cell lymphoma

A genome‐wide CRISPR/Cas9 knockout screen identifies critical genes influencing malignant T‐cell susceptibility to DMF. The T‐ALL cell line CEM was selected as a cell model screening system due to its higher transduction efficiency, which is crucial for reliable gene‐editing outcomes. In contrast, the Sezary cell line HH exhibited markedly lower transduction efficiency, potentially limiting the sensitivity and effectiveness of the CRISPR screen (Fig. S1a) We established a stable Cas9 overexpressing CEM cell line using lentiviral transduction, selection, and single‐cell sorting. Cas9 expression was validated by western blot analysis (Fig. S1b). To confirm Cas9 functionality, CEM‐Cas9 cells were transduced with sgRNA targeting a model protein (here CD95). Flow cytometry showed a significant decrease in CD95 surface expression, confirming effective Cas9‐mediated gene knockout (Fig. S1b,c).

**Fig. 1:**
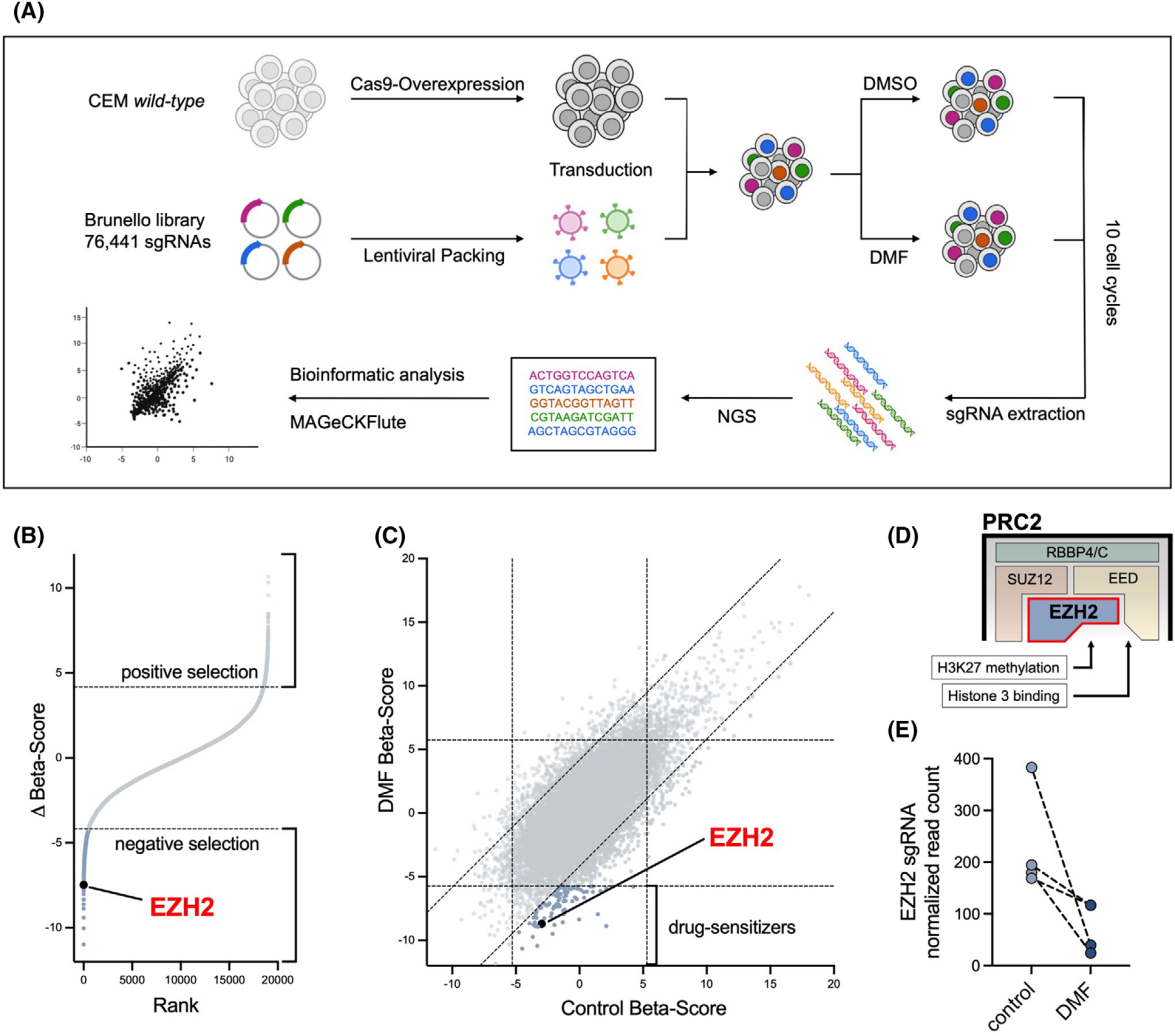
A genome‐wide CRISPR/Cas9 knockout screen identifies enhancer of Zeste Homolog 2 (EZH2) as a critical gene in DMF treatment. (A) Schematic overview of a genome‐wide CRISPR/Cas9 knockout screen. The Brunello single‐guide RNA (sgRNA) library containing 76 441 sgRNAs was lentivirally packaged and transduced into Cas9‐overexpressing CEM cells. Cells were incubated with dimethyl fumarate (DMF) or vehicle control (DMSO) for 10 estimated cell cycles (12 days). SgRNAs were amplified by PCR and quantified by Next Generation Sequencing (NGS). Datasets were analyzed using the MAGeCKFlute algorithm. (B) EZH2 was identified among the negatively selected genes in the DMF‐treated group. Beta score is calculated by MAGeCK‐Flute and represents fold‐changes. (C) Square plot analysis revealed EZH2 as a drug sensitizer gene. Drug‐sensitizing genes are those whose targeting leads to an isolated proliferation inhibition in the DMF‐treated group. In the untreated group, drug sensitizer genes exhibit no altered proliferation. (D) EZH2 is the catalytic center of the Polycomb Repressive Complex 2 (PRC2), mediating histone H3 binding and H3K27 methylation. (E) The normalized read count of all EZH2‐targeted sgRNAs after 10 cell cycles. The lines show the corresponding sgRNAs in both groups. Subfigure A was created using biorender.com. *n* = 2 biologically independent samples in panel B, C and E.

After establishing Cas9‐expressing CEM cells, we performed lentiviral transduction with the Brunello library targeting 19 114 genes, requiring a minimum of 9 × 10^7^ cells per sample to achieve 400x library coverage. Cells were split into DMF‐treated and control groups, incubated for approximately 10 cell cycles (12 days). SgRNAs were extracted via PCR, sequenced using NGS, and analyzed with the MAGeCKFlute algorithm (Fig. 1A) [28]. The data include raw sgRNA counts, revealing the representation of specific cell subtypes. Cells with sgRNAs targeting DMF‐sensitizing genes were underrepresented after treatment. We identified 112 genes significantly negatively selected in the DMF‐treated group compared to controls (Fig. 1B). To avoid bias from genes influencing general cell viability, we excluded those altered in the untreated group. Only genes that impacted survival and proliferation in the DMF‐treated group were considered potential drug sensitizers (Fig. 1C).

Among the drug sensitizers, we discovered the Enhancer of Zeste Homolog 2 (EZH2) which serves as the catalytic core of the Polycomb Repressive Complex 2 (PRC2). EZH2 is responsible for the mono‐, di‐, and tri‐methylation of lysine 27 on histone H3 (H3K27me1/2/3), functioning as a transcriptional regulator (Fig. 1D) [29, 30]. EZH2 is a well‐established and clinically relevant therapeutic target in several malignancies, including T‐ and B‐cell lymphomas [31, 32]. All sgRNAs targeting EZH2 were strongly underrepresented in the DMF‐treated group, suggesting that loss of EZH2 enhances DMF treatment efficacy (Fig. 1E).

### EZH2 downregulation confirms its role in enhancing DMF‐induced cell death in T‐cell lymphoma

**Fig. 2:**
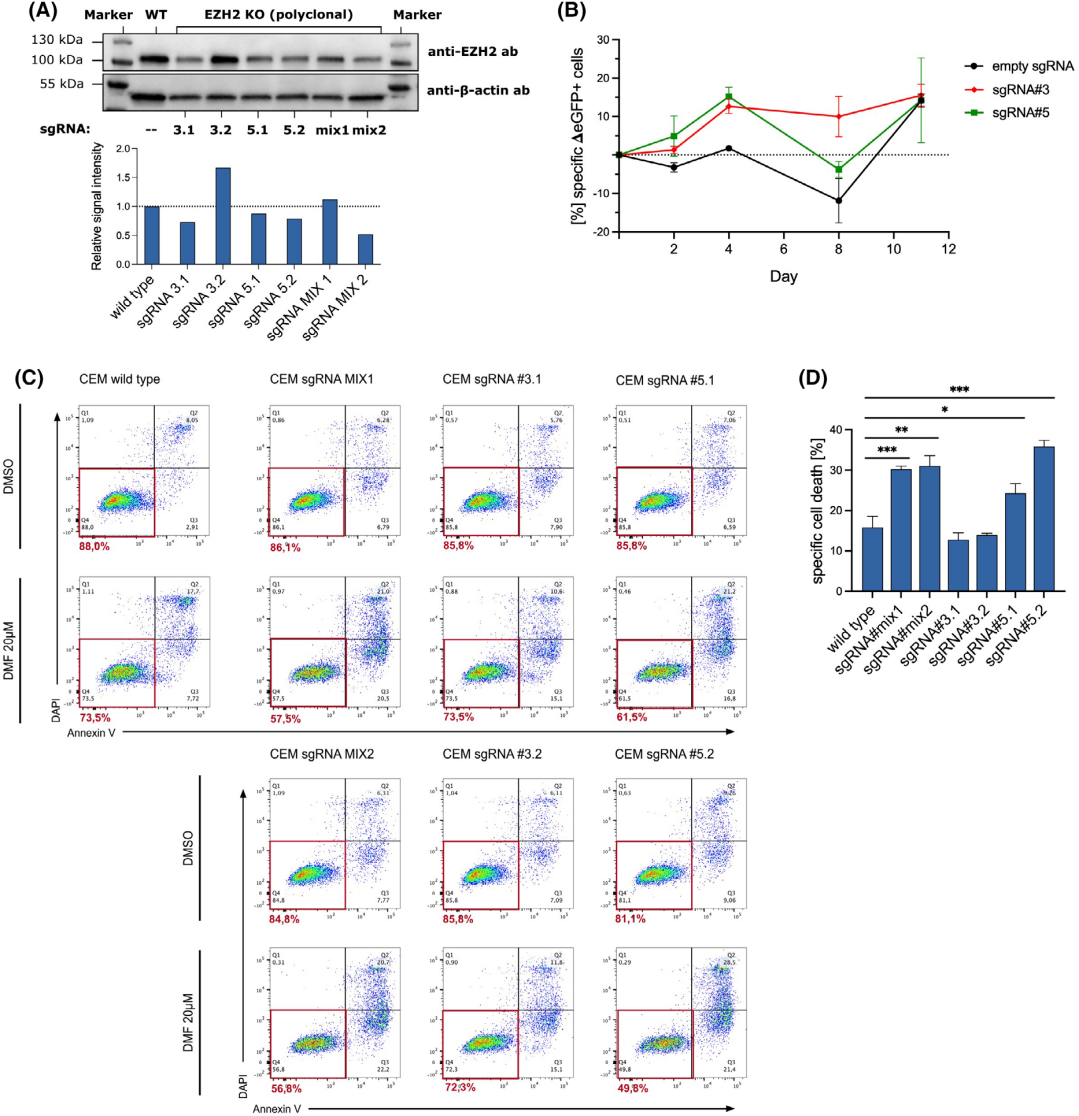
Single‐clone knockdown confirms EZH2 as a gene sensitizing toward DMF treatment. (A) Western blot analysis of CEM‐sgRNA cells. The upper panel depicts EZH2 expression, while the lower panel illustrates beta‐Actin expression, utilized as a loading control. (B) Competition assay evaluated the relative proliferation of CEM‐sgRNA and CEM cells with an empty sgRNA backbone compared to cocultured CEM‐eGFP+ cells. The amount of GFP+ cells was determined on day 0, 2, 4, 8 and 11. Specific changes in the frequency of eGFP+ cells in response to DMF treatment were displayed. (C) Cell death assay for CEM wild‐type and CEM‐sgRNA cells treated with DMSO or DMF, incubated for 48 h. Analyzed using flow cytometry with Annexin V/DAPI staining. The Annexin V‐/DAPI‐ population represents viable cells and is indicated with a red square. (D) Quantification of cell death determined from the Annexin V/DAPI assay in panel C. Comparison of CEM *wild‐type* and CEM‐sgRNA cells. Specific cell death was calculated. The error bar in panels B and D represents the standard deviation (SD). *n* = 3 biologically independent samples. Student's *t*‐test *P < 0.05, **P < 0.01, ***P < 0.001.

A single‐clone experiment was conducted using two different EZH2‐targeting sgRNAs to validate the screen hits. To enhance downregulation efficiency, mixed clones were generated by incorporating both sgRNAs. To minimize potential confounding factors associated with the Cas9‐overexpressing CEM clone, we used a lentiviral vector that co‐delivers both the Cas9 and the sgRNA sequences (Fig. S2a). Equal amounts of lentiviral units carrying sgRNA#3 and #5 were used to generate the sgRNA#mix clones. CEM *wild‐type* cells were transduced and subsequently processed following the identical protocol used in the screening experiment. To assess the efficacy of downregulation, we performed western blot analysis on two clones for each sgRNA. Notably, sgRNA#3, sgRNA#5, and sgRNA#mix resulted in a modest downregulation of EZH2 expression. This implies that under screening conditions, the sgRNA‐mediated DNA double‐strand breaks were not efficient enough to achieve a complete knockout. However, a discernible reduction in EZH2 expression was observed (Fig. 2A). To investigate the proliferation of EZH2‐downregulated CEM cells (CEM‐sgRNA) in both untreated and DMF‐treated conditions, we conducted a competition assay. CEM‐sgRNA#3 and #5 clones and CEM cells carrying an empty sgRNA backbone were used, and each sample was combined in a 1 : 1 ratio with stable overexpressing CEM‐GFP cells. Cells were treated with 18 μm DMF. The proportion of CEM‐GFP^+^ cells was monitored by flow cytometry over a period of 2 to 11 days. Assuming a uniform proliferation of GFP‐positive cells in all samples, the ratio of GFP‐positive cells to GFP‐negative cells provides information on the survival and thus the sensitization of the cells to DMF treatment. This is achieved by assessing the proliferation of each cell line relative to the baseline proliferation of GFP‐expressing CEM cells. The relative changes of GFP‐positive cells were determined under untreated and DMF‐treated conditions (Fig. S2b–d). The DMF‐dependent changes in the amount of GFP‐positive cells in the samples were then calculated. In comparison to CEM‐empty sgRNA controls, DMF treatment resulted in a relative increase in GFP‐positive cells within the sgRNA#3 and sgRNA#5 CEM samples. This suggests a proliferative disadvantage of EZH2‐downregulated cells under treatment conditions.(Fig. 2B). To explore the impact of EZH2 downregulation on DMF‐mediated cell death induction, we conducted an Annexin V/DAPI‐based apoptosis assay using flow cytometry. Under untreated conditions, all knockdown cell lines demonstrated comparable viability to wild‐type cells. Of note, CEM‐sgRNA#5 and CEM‐sgRNA#mix cells exhibited notably elevated levels of cell death induced by DMF, reaching up to 15.6% additional specific cell death (Fig. 2C,D), indicating that the downregulation of EZH2 sensitizes CEM cells toward DMF treatment.

In summary, we were able to validate our screening results. We demonstrated that targeting EZH2 does not affect the cell viability of untreated cells but increases cell sensitivity and induction of cell death toward DMF treatment.

### EZH2‐inhibitor tazemetostat sensitizes T‐ALL and CTCL cell lines to DMF treatment

**Fig. 3:**
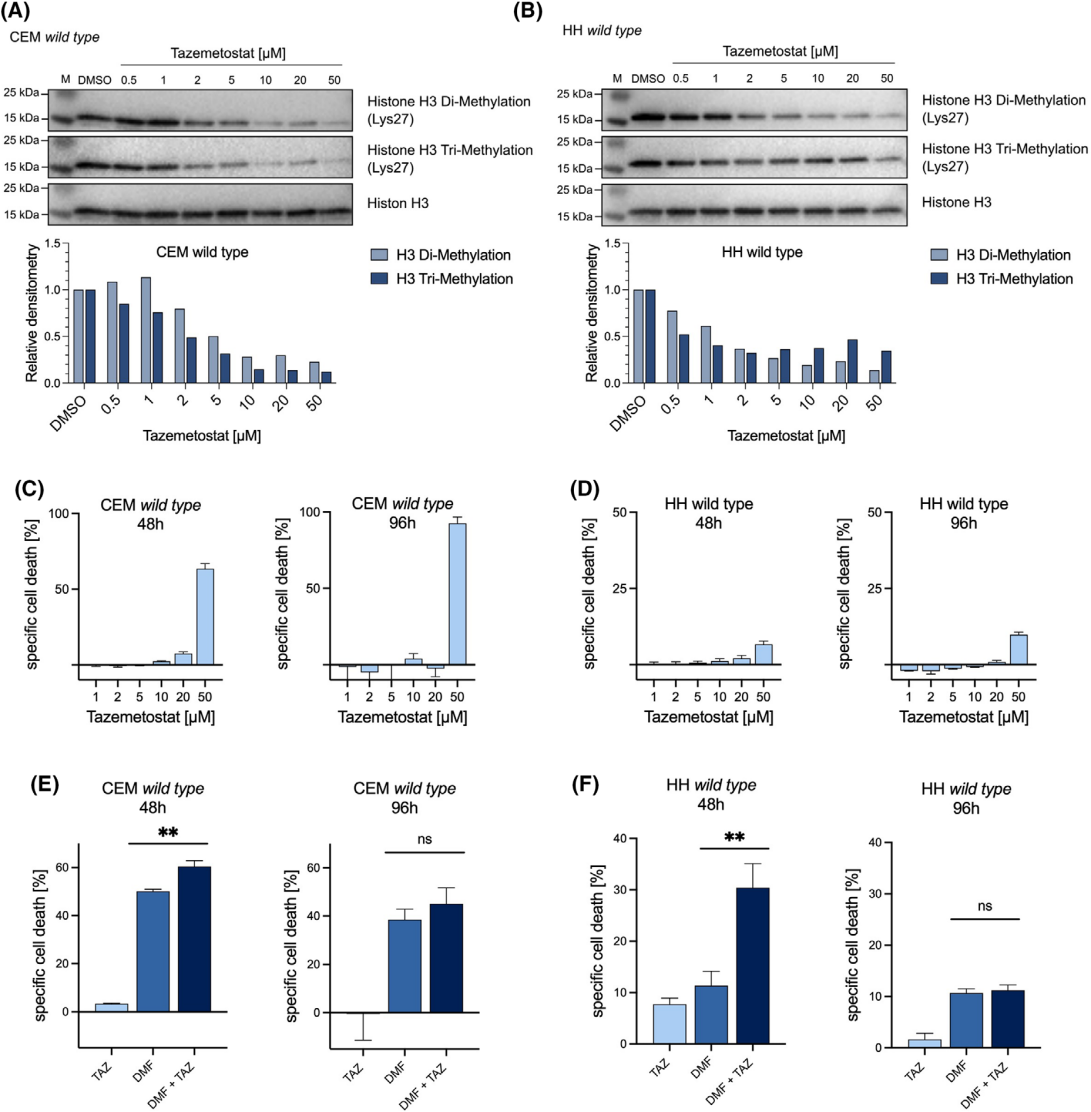
EZH2‐inhibitor tazemetostat sensitizes CEM and HH wild‐type cells for DMF treatment. (A, B) Western blot analysis of di‐ and tri‐methylation of Lysine 27 on histone H3 (H3K27) in CEM and HH cells 48 h after tazemetostat treatment. Tazemetostat concentrations ranging from 0.5 to 50 μm were employed. The upper panel shows di‐methylated H3K27. The center panel shows tri‐methylated H3K27. The lower panel shows the amount of Histone 3, used as a loading control. Densitometric analysis is shown below. (C, D) Specific cell death of CEM and HH *wild‐type* cells treated with tazemetostat. Conducted via flow cytometry‐based Annexin V/DAPI staining. Cell death was determined 48 h and 96 h after tazemetostat treatment. Increasing tazemetostat concentrations from 1 to 50 μm were utilized. (E, F) Specific cell death of CEM and HH *wild‐type* cells treated with DMF and tazemetostat (TAZ). Conducted via flow cytometry‐based Annexin V/DAPI staining. Cells were treated with DMF (25 μm), tazemetostat (5 μm), and a combination of DMF and tazemetostat (25 μm/5 μm). Cell death was determined after 48 h and 96 h. Specific cell death was calculated. The error bars represent the standard deviation (SD). *n = 3* biologically independent samples. Student's *t*‐test ns, not significant, **P < 0.01.

Tazemetostat is an FDA‐approved small molecule that specifically inhibits the enzymatic function of EZH2 [33]. To further explore the potential of combined treatment with DMF and EZH2 targeting compounds, we investigated the effect of tazemetostat on DMF‐induced cell death in CEM cells, a T‐ALL cell line, as well as in Sézary syndrome‐derived HH cells. Various studies have used different concentrations of Tazemetostat for different cell lines (0.5–20 μm) [34, 35, 36, 37]. To identify the ideal treatment concentration for HH and CEM cells, we conducted a systematic titration of Tazemetostat. Cells were subjected to various Tazemetostat concentrations (0.5–50 μm), and the resulting inhibition of EZH2 and subsequent H3K27 methylation was quantified by western blot and densitometric analysis. After 48 h in both CEM and HH cells, a major decrease of di‐ and tri‐methylation was observed with increasing concentrations, confirming robust inhibition of EZH2 and PRC2 function (Fig. 3A,B). Of note, this effect remained at a comparable level after 96 h, suggesting that a 48 h incubation is sufficient to achieve stable inhibition of EZH2 function (Fig. S3a,b). Since our data indicated that loss of EZH2 expression alone did not affect cell viability, we investigated the cell death‐inducing effect of tazemetostat monotherapy on CEM and HH cells. The cells were exposed to increasing concentrations of tazemetostat (1–50 μm) and incubated for 48 and 96 h. The analyses revealed no influence on cell viability at concentrations below 10 μm in CEM (Fig. 3C) and below 50 μm in HH cells (Fig. 3E). Remarkably, no increase in cell death was observed even after 96 h, further supporting the tolerability of lower tazemetostat concentrations. Since the suppression of H3K27 tri‐methylation is detectable at significantly lower concentrations, the cell death‐inducing effect of Tazemetostat observed at higher concentrations does not appear to be related to inhibition of EZH2 activity. Considering our data set, we determined for 5 μm Tazemetostat to be the optimal treatment concentration for CEM and HH cells to achieve a significant inhibition of EZH2 activity without a notable impact on cell viability.

To assess the potential sensitizing effects of tazemetostat in DMF treatment, we conducted a combination treatment experiment using unmodified CEM and HH cells. The combination treatment effectively increased the effects of DMF in both cell lines. In the CEM cell line, tazemetostat significantly increased DMF‐induced specific cell death by up to 10% (Fig. 3D). Of note, these findings showed an even more pronounced effect in HH cells, where tazemetostat more than doubles the DMF‐mediated induced specific cell death (Fig. 3F). To assess the stability of this effect over time, specific cell death was additionally measured after 96 h. Interestingly, the combinatorial effect observed at 48 h could no longer be detected at this later time point, suggesting that the sensitizing effect of tazemetostat is transient and manifests predominantly within the first 48 h of treatment (Fig. 3D,F).

In summary, we demonstrated that tazemetostat, when administered alone, effectively inhibits EZH2 activity, without adversely affecting cell viability. Moreover, in conjunction with DMF, it induces a highly significant increase in cell death. These findings prove that EZH2 inhibition sensitizes both CEM and HH cells to DMF treatment.

### Loss of PRC2 function is a key factor in sensitization toward DMF treatment

**Fig. 4:**
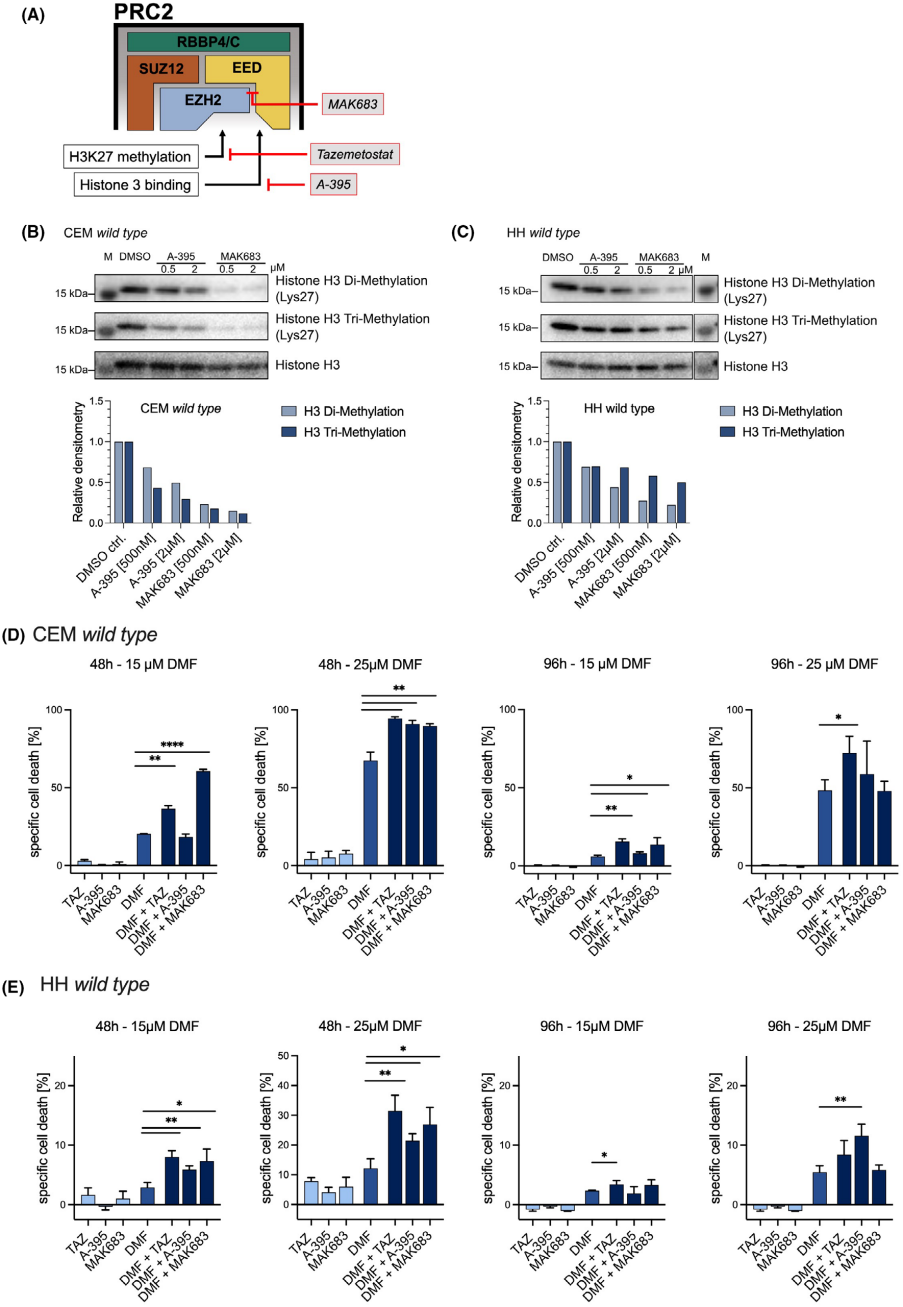
Inhibition of H3K27me3 is crucial for tazemetostat‐mediated DMF sensitization. (A) Schematic overview of PRC2 core units and the used inhibitors. Tazemetostat acts as antagonists to the EZH2 catalytic core. A‐395 is a specific inhibitor of the Embryonic Ectoderm Development (EED)‐H3K27 binding unit. MAK683 prevents EZH2 – EED interaction and subsequently the formation of PRC2. (B, C) Western blot analysis of di‐ and tri‐methylation of Lysine 27 on histone H3 (H3K27) in CEM and HH cells treated with different concentrations of A‐395 (0.5/2 μm) and MAK638 (0.5/2 μm). The upper panel shows the amount of di‐methylated H3K27. The center panel shows the amount of tri‐methylated H3K27. The lower panel shows the amount of Histone 3, used as a loading control. Densitometric analysis is shown below. (D, E) Specific cell death of CEM and HH *wild‐type* cells treated with DMF and PRC2 inhibitors was conducted via flow cytometry‐based Annexin V/DAPI staining. Visualized apoptosis assay of CEM *wild‐type* cells. Cells were treated with tazemetostat (5 μm), A‐395 (0.5 μm), or MAK683 (0.5 μm) in combination with a DMSO control or DMF (25 μm). Cell death induction was determined after 48 h and 96 h using a flow cytometry‐based Annexin V/DAPI staining. Specific cell death was calculated. The error bars represent the standard deviation (SD). *n = 3* biologically independent samples. Student's *t*‐test *P < 0.05, **P < 0.01, ****P < 0.0001.

It is well established that EZH2 serves as the catalytic core of the PRC2, mediating the methylation of Lysine 27 on Histone H3 (H3K27me). In addition, many studies have shown that EZH2 also exerts effects independent of PRC2, influencing gene activation, cancer progression, and immune modulation [38, 39, 40]. To further investigate whether the observed effects of EZH2 inhibition were due to the absence of PRC2 activity, we examined the effects of various PRC2 inhibitors on DMF treatment. PRC2 consists of four major subunits: Enhancer of Zeste Homolog 2 (EZH2), Embryonic Ectoderm Development (EED), Suppressor of Zeste 12 (SUZ12), and Retinoblastoma Binding Protein 4 (RBBP4) or Retinoblastoma Binding Protein 7 (RBBP7). EZH2 functions as the catalytic core, with EED and SUZ12 serving as regulatory subunits that stabilize the complex and augment its histone methyltransferase activity. In addition, RBBP4/RBBP7 plays a role in target recognition and binding. Since EED methyltransferase activity is crucial for PRC2 function, we examined the effect of the EED inhibitors A‐395 and MAK683. While A‐395 specifically antagonizes the H3K27 binding functions of EED [41], MAK683 inhibits the EED–EZH2 interaction, which prevents the formation of PRC2 in general (Fig. 4A) [42].

The suppression of H3K27me was determined by western blot and subsequently quantified using densitometric analysis. In both cell lines, the inhibitors demonstrated sufficient blockage of H3K27me after 48 h. Consequently, increasing the concentration does not further reduce H3K27me levels (Fig. 4B,C). In this case as well, the effect could not be observed to a greater extent after 96 h (Fig. S4a,b).

We extended our investigation of the DMF‐sensitizing effects by analyzing cell death induction under varying treatment conditions. Consistent with previous results, individual inhibitors and PRC2 silencing did not affect cell viability. When combined with DMF, however, all tested PRC2 inhibitors enhanced DMF‐mediated cell death. After 48 h, both 15 and 25 μm DMF treatment in combination with the PRC2 inhibitors led to a significant increase in cell death in both CEM and HH cells, although the overall cell death induction was lower with 15 μm DMF (Fig. 4D,E). In particular, we observed a pronounced sensitizing effect in HH cells at 25 μm DMF, where the combination treatment nearly doubled DMF‐induced cell death (Fig. 4E). After 96 h, the overall levels of induced cell death were markedly reduced, consistent with the known time‐limited cytotoxicity of DMF. Consequently, the sensitizing effects of PRC2 inhibition were also significantly diminished in both cell lines and failed to reach statistical significance in several conditions.

Taken together, these data confirm that pharmacological inhibition of PRC2—regardless of the specific molecular target—enhances the cytotoxic effects of DMF most effectively within the first 48 h of treatment. These findings underscore the therapeutic potential of combining DMF with PRC2 inhibition and highlight the importance of timing in maximizing treatment efficacy.

### PRC2‐targeting augments DMF‐induced cell death specifically in primary Sézary cells

**Fig. 5:**
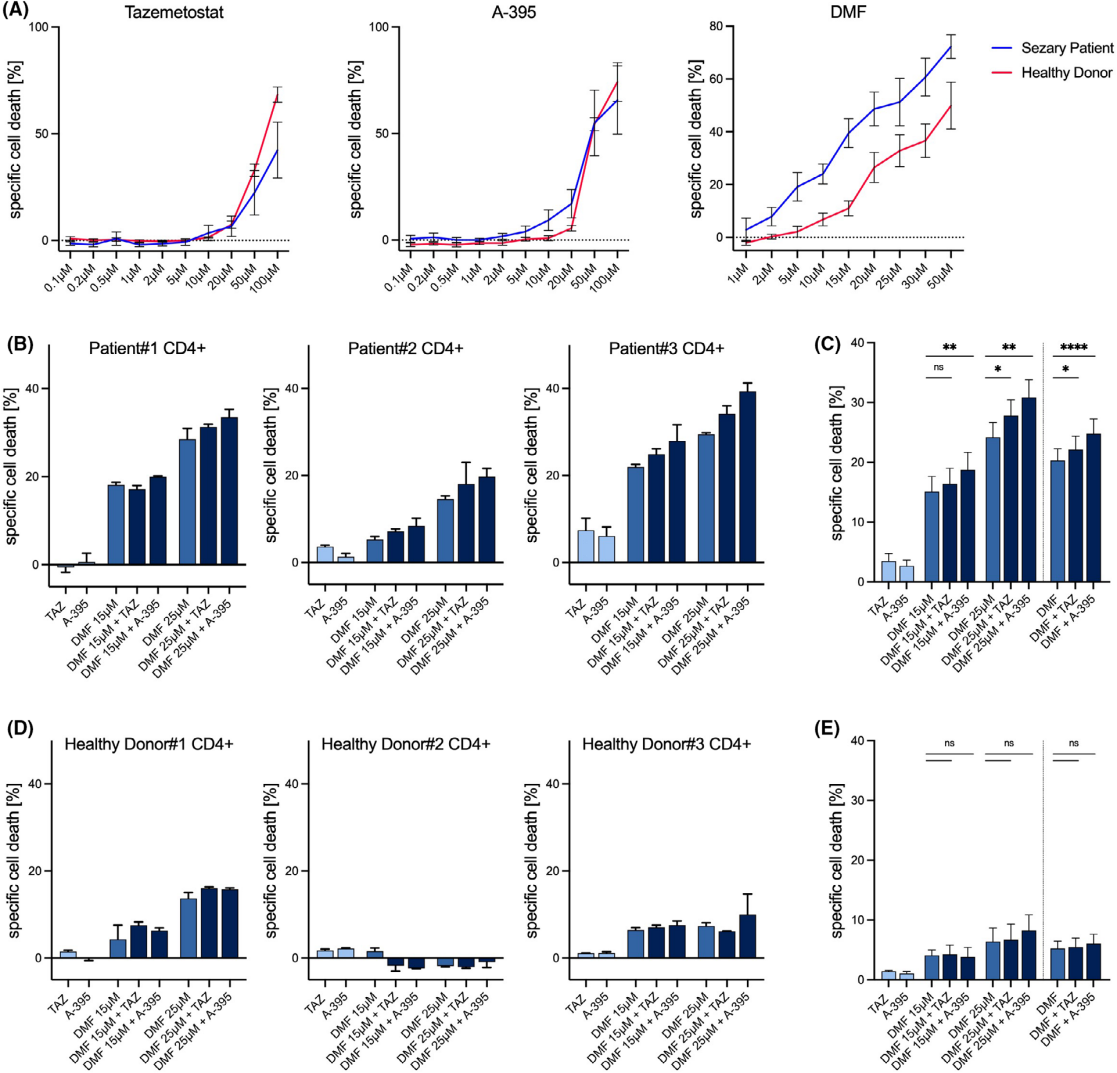
Combination treatment specifically sensitizes patient‐derived Sézary cells to DMF treatment. (A) Dose‐response curves of tazemetostat, A‐395, and DMF in primary CD4+ cells derived from Sézary syndrome and healthy donors. Cells were treated with increasing concentrations as indicated below. Cell death was determined using a flow cytometry‐based Annexin V/DAPI staining. (B) Specific cell death of primary CD4+ cells derived from Sézary syndrome patients treated with DMF and PRC2 inhibitors. Treatments included either DMF (15/25 μm), tazemetostat (5 μm), and A‐395 (0.5 μm) alone, or as combination treatments in different concentrations. Cell viability was determined after 48 h using flow cytometry‐based Annexin V/DAPI staining. Specific cell death was calculated. (C) Summary of specific cell death induction by various treatment combinations across all analyzed patient‐derived samples. Cells were treated with DMF and PRC2 inhibitors as indicated. The last section summarizes all DMF‐treated samples (15 μm and 25 μm). (D) Specific cell death of primary CD4+ cells derived from healthy donors treated with DMF and PRC2 inhibitors. Treatments included DMF (15/25 μm), tazemetostat (5 μm), and A‐395 (0.5 μm) in various combinations, as indicated below. Cell viability was determined after 48 h using a flow cytometry‐based Annexin V/DAPI staining. Specific cell death was calculated. (E) Summary of specific cell death induction by various treatment combinations across all analyzed healthy donor‐derived samples. Cells were treated with DMF and PRC2 inhibitors as indicated. The last section summarizes all DMF‐treated samples (15 μm and 25 μm). The error bar represents the standard deviation (SD) in panels A, B, and D. The error bar in panels C and E represents the SEM. *n* = 3 biologically independent samples in panels A, B, and D. n = 9 biologically independent samples in panels C and E. Wilcoxon‐Test: ns, not significant, *P < 0.05, **P < 0.01, ****P < 0.0001.

To evaluate the potential benefits of combined drug treatment in a more clinical setting, we performed experiments using primary cells from Sézary patients and healthy donors. By using patient‐derived cells, our study aimed to better understand the efficacy and relevance of the dual‐treatment strategy for this specific patient group, given the variability in immunophenotype and gene expression between individuals. In addition, we included experiments with cells from healthy donors to determine whether the effects were specific to malignant cells. Primary CD4^+^ T cells were isolated from peripheral blood and exposed to increasing concentrations of DMF, tazemetostat, or A‐395. While malignant T cells displayed a markedly enhanced susceptibility to DMF‐induced cell death compared to healthy CD4^+^ T cells, this selective sensitivity was not observed with either tazemetostat or A‐395. Both PRC2 inhibitors induced comparable levels of cell death in malignant and non‐malignant T cells, underscoring the tumor‐specific cytotoxic potential of DMF (Fig. 5A). Next, primary CD4^+^ cells were treated with DMF and either Tazemetostat or A‐395. We observed that the combination treatment of DMF plus Tazemetostat and DMF plus A‐395 enhanced the induction of cell death in patient‐derived CD4^+^ cells. The combination of A‐395 demonstrated the ability to significantly enhance DMF‐induced cell death, increasing it by more than 34% in cells derived from patient #3 (Fig. 5B). Despite the variability in effects between different patients, an overall significant increase of induced cell death was observed in patient‐derived cells treated with DMF and a PRC2 inhibitor. Our analyses confirm that both inhibitors significantly enhance DMF‐induced cell death in malignant T cells. However, we did not observe a stronger synergistic effect at the lower DMF concentrations (15 μm) compared to higher doses (25 μm) (Fig. 5C).

Interestingly, this sensitizing effect is not observed in cells derived from healthy donors. In addition to the significantly lower induction of cell death by DMF, no enhancement of cell death induction by tazemetostat or A‐395 can be detected (Fig. 5D,E).

In summary, we have successfully demonstrated the capability of PRC2 inhibition to enhance DMF‐mediated cell death induction in patient‐derived cells. Furthermore, we have shown that this effect does not occur in cells from healthy donors. This suggests a high specificity for malignant cells. These findings underscore the potential promise of combined treatment with DMF and PRC2 inhibitors in future therapeutic strategies.

## Discussion

The Sézary syndrome, an aggressive and incurable variant of CTCL, presents a significant challenge in hematological malignancies due to its resistance to therapy and variability in clinical presentation and progression [43, 44]. Despite notable advances in the treatment of CTCL, for example, through the use of brentuximab [45], a CD30 antibody, and mogamulizumab [46], there is an urgent need for novel treatment options [47].

DMF, a small molecule targeting NF‐κB, has shown promising results in a recent phase II clinical trial [11]. The efficacy of DMF is comparable to that of brentuximab or mogamulizumab, with the advantage of fewer side effects [11]. Despite the positive results, the need for further improvement of DMF therapy has become apparent due to cases of limited or suboptimal treatment response [11]. Targeted therapies are often limited by decreased efficacy, resulting from microenvironmental adaptations and compensatory pathway activation [44, 48, 49, 50]. Here, we conducted a genome‐wide CRISPR/Cas9 knockout screening to identify EZH2, a key component of the Polycomb Repressive Complex 2 (PRC2), as a critical regulator of DMF‐induced cell death. We propose PRC2 as a novel target to augment the anticancer efficacy of DMF in malignant T‐cell populations.

Genome‐wide CRISPR screenings are a well‐established and robust platform that empowers researchers to gain profound insights into genetic networks and relationships [51, 52, 53]. We employed the Brunello sgRNA knockout library, providing 76 441 sgRNA sequences targeting 19 114 genes. The library has previously demonstrated robust results in whole‐genome knockout screens [54, 55]. We assessed the quality of the screen by evaluating read depth, sgRNA completeness, and coverage. Our analysis identified EZH2 as a promising target for DMF sensitization, evidenced by a significant depletion of EZH2 knockout cells upon DMF treatment. Validation with EZH2 knockout clones confirmed increased sensitivity to DMF, showing reduced proliferation and elevated cell death compared to wild‐type cells. Importantly, in the absence of DMF, EZH2 knockout clones exhibited no deficits in viability or proliferation, highlighting the specific DMF‐sensitizing effect of EZH2 targeting in CEM T cell lines.

The FDA‐approved EZH2 inhibitor tazemetostat, designed specifically for the treatment of prostate carcinoma, is a SAM‐competitive inhibitor that binds to the catalytic pocket of EZH2, blocking its methyltransferase activity [56]. Our experiments involved the use of tazemetostat in combination with DMF in CEM and HH cells, which mirrored findings in EZH2 knockout cells. We demonstrated that EZH2 inhibition by Tazemetostat significantly enhanced DMF‐mediated cell death induction, suggesting that Tazemetostat may act as a sensitizer to DMF treatment.

The PRC2 consists of four major protein subunits mediating complex integrity, target recruiting, and histone methylation. EZH2 is the core unit of PRC2 and catalyzes the tri‐methylation of lysine 27 on histone H3 (H3K27me3) [57]. By using different inhibitors that affect different aspects of PRC2 complex formation and function, we were able to show that the effects observed with EZH2 targeting are due to PRC2 dysfunction and not to an alternative PRC2‐independent pathway of EZH2. Our findings strongly suggest that the methylation status of lysine 27 on histone H3 is a critical determinant of the efficacy of DMF‐mediated cell death induction. Genome‐wide analyses have revealed that EZH2 and NF‐κB factors co‐localize at specific genomic sites and, in a subset of cases, EZH2 mediates the positive regulation of NF‐κB target genes [58]. The resulting activation of select NF‐κB targets supports oncogenic programs, including enhanced migration and the acquisition of stemness phenotypes, thereby contributing to cancer progression. In this context, overexpression of EZH2 may sustain or even boost NF‐κB signaling.

DMF treatment, an NF‐κB‐inhibiting, cell death‐inducing therapy, represents a significant advancement for Sézary syndrome, where treatment options remain limited. We demonstrated that DMF induces cell death in Sézary T‐cell lines and significantly reduces tumor growth and metastasis in CTCL xenograft mouse models [5, 59]. Our previous *in vitro* experiments showed that DMF reduces NF‐κB activity by inactivating Thioredoxin‐1 via monomethyl succinylation, leading to decreased expression of antiapoptotic IAP proteins (XIAP, cIAP1, cIAP2) and formation of the ripoptosome, inducing apoptotic or necroptotic cell death [6]. Given that both EZH2 inhibition and DMF therapy interfere with NF‐κB signaling through distinct mechanisms, their combination may synergistically repress NF‐κB‐driven survival pathways and restore treatment sensitivity in malignant T cells. EZH2 has been shown to modulate NF‐κB activity both indirectly—by repressing genes involved in redox regulation, immune response, and apoptosis—and directly, through non‐canonical interactions with NF‐κB subunits [60, 61]. Conversely, NF‐κB signaling has also been implicated in the transcriptional regulation of EZH2, indicating a bidirectional crosstalk [62]. Thus, dual targeting of these pathways may disrupt reinforcing survival mechanisms and enhance therapeutic efficacy.

DMF also generates reactive oxygen species (ROS), causing oxidative damage to DNA, proteins, and lipids [63, 64]. There is evidence that PRC2, in turn, controls the expression of genes involved in the response to oxidative stress [65]. For instance, by controlling genes in the NRF2 pathway or those encoding antioxidant enzymes, EZH2 activity might help cancer cells better cope with DMF‐induced oxidative stress. Inhibiting PRC2 may lead to a reduced threshold of DMF‐induced cell death. While no studies to date have directly investigated the combination of PRC2 inhibitors with DMF, the mechanistic link between epigenetic regulation and redox homeostasis provides a compelling rationale for this therapeutic approach.

Recently, we confirmed the clinical efficacy of DMF in a phase II clinical trial [11]. However, the limitations of mono‐target therapies are evident from the isolated cases of poor responses and loss of efficacy observed in the trial [11]. Our study demonstrated that inhibition of PRC2 significantly increased the sensitivity of primary Sézary cells to DMF‐induced cell death, with little or no effect on CD4+ cells derived from healthy donors. This suggests that combination therapy may boost DMF efficacy, address limited responsiveness in Sézary syndrome, and improve clinical outcomes with minimal toxicity. PRC2 and H3K27me3 are epigenetic regulators that modulate gene expression by altering chromatin structure and limiting DNA accessibility [57]. Targeting EZH2 and PRC2 has emerged as a promising strategy for treating various cancer entities, with several ongoing clinical trials investigating different EZH2 or PRC2 inhibitors [30, 66]. *In vitro* experiments have demonstrated that EZH2 inhibitors can induce cell death through multiple mechanisms [67, 68]. However, the precise mechanism underlying EZH2 targeting‐mediated cell death remains elusive.

Nevertheless, recent studies have provided a more detailed description of certain aspects of these mechanisms. Notably, it has been revealed that PRC2 and H3K27me3 can play a pivotal role in the DNA repair processes [69]. DNA repair mechanisms are crucial in response to oxidative stress. Suppressing these functions may explain the enhanced efficacy of ROS‐inducing therapies like DMF treatment [70].

In chronic myeloid leukemia cells, Nishoka *et al*. demonstrated that EZH2 directly influences the expression of XIAP and Bcl‐2 through the activation of microRNAs. Targeting EZH2 with shRNA significantly reduced the miR‐219‐mediated expression of the antiapoptotic XIAP and Bcl‐2 [71]. Interestingly, it has also been demonstrated that treatment with the tyrosine kinase inhibitor Imatinib leads to the activation of STAT5 in these cells, resulting in the upregulation of EZH2 [72]. This implies that the overexpression of EZH2 in these cells is utilized as a cellular strategy to counteract apoptosis induction caused by imatinib.

This correlation may explain the sensitizing effects of EZH2 inhibition in DMF treatment, as DMF‐induced cell death involves NF‐κB‐mediated suppression of XIAP and BCL‐2. These findings align with our previous study showing synergy between DMF and the BCL‐2 inhibitor ABT‐199 [59]. Supporting this hypothesis, targeting EZH2 may enhance antiapoptotic signals, explaining the sensitization of malignant T cells to DMF therapy.

While other studies have suggested that EZH2 may mediate oncogenic effects in prostate cancer through PRC2‐independent activation of the STAT3 pathway [38], our experiments have ruled out this axis as we demonstrated that the observed effects belong to the inhibition of PRC2. The precise relationship between H3K27me inhibition and DMF sensitization remains unclear, requiring further research. Epigenetic modifications are emerging as a promising strategy to enhance anticancer drug efficacy [73, 74] Our study confirmed that PRC2 inhibition enhances DMF effectiveness via epigenetic modulation. This approach may extend to other cancers with limited DMF response, such as colorectal carcinoma, where DMF‐induced cell death and EZH2 inhibition may promote apoptosis [75, 76].

Our whole‐genome CRISPR/Cas9 screen identified genes influencing DMF‐mediated cell death. We confirmed that H3K27me inhibition enhances DMF efficacy, establishing the first link between epigenetic modification and DMF‐induced cell death. This suggests a novel strategy of combining DMF with sensitizing co‐treatments to overcome the limitations of DMF therapy.

## Material and methods

### Cell culture

CEM (ATCC; CCL‐119) and HH cell lines (ATCC; CRL‐2105) were cultured in RPMI medium (Gibco, Thermo Fisher Scientific, Dreieich, Germany) supplemented with 10% fetal bovine serum and 100 μg ml^−1^ penicillin–streptomycin and cultured at 37 °C and 5% CO_2_. HEK293T cells (ATCC; CRL‐3216) were cultured in DMEM (Gibco, Thermo Fisher Scientific) supplemented with 10% fetal bovine serum (anprotec, Fetal Clone II) and 100 μg ml^−1^ penicillin–streptomycin (Thermo Fisher Scientific) and cultured at 37 °C and 10% CO_2_.

All cell lines used in this study were obtained directly from the American Type Culture Collection (ATCC) and were maintained according to the supplier's recommendations. Cells were routinely tested for mycoplasma contamination using the PlasmoTest™ (InvivoGen, San Diego, CA, USA), and only mycoplasma‐free cultures were used for experiments. To confirm cell line identity, short tandem repeat (STR) profiling was conducted during the study at the Leibniz Institute DSMZ—German Collection of Microorganisms and Cell Cultures.

### Lymphocyte separation

Human peripheral blood leukocytes were purified as described [4] Then, T cells were sorted with the CD4+ T Cell Isolation Kit II (Miltenyi Biotec, Bergisch Gladbach, Germany). The study followed DKFZ ethical guidelines and the Helsinki Declaration and was approved by the Ethics Committee II of Heidelberg University (2018‐653N‐MA). Peripheral blood samples were collected between January 2023 and March 2025. Samples from healthy donors were obtained either at the Department of Internal Medicine III (Hematology and Oncology), University Hospital Regensburg, Regensburg, Germany, or at the Department of Dermatology, Venereology and Allergology, University Medical Center Mannheim, University of Heidelberg, Mannheim, Germany. All patient samples were collected at the Department of Dermatology, Venereology and Allergology, University Medical Center Mannheim, University of Heidelberg, Mannheim, Germany. All blood donors provided written informed consent agreeing that the collected samples may be used for research purposes. Cells were cultured in RPMI with 10% fetal bovine serum (anprotec, Fetal Clone II).

### Lentivirus production and titration

An sgRNA‐carrying lentiviral plasmid, combined with third‐generation packaging plasmids (pRSV‐Rev71, pMDLg/pRRE71, pCMV‐VSVg), was transfected into HEK293T cells via lipofection (X‐treme GENE HP, Roche, Basel, Switzerland). Lentiviral particles were concentrated using Lenti‐X™ Concentrator (Takara Bio, Saint‐Germain‐en‐Laye, France) according to the manufacturer's protocol and stored at −80 °C. Concentrations were determined using the Lenti‐X p24 Rapid Titer Kit (Takara Bio). Transduction efficacy was assessed using eGFP‐carrying lentiviruses titrated to CEM. Transfected cells were quantified after 48 h by flow cytometry (LSR‐Fortessa, BD Biosciences, Heidelberg, Germany), and the infectious units for a specific MOI were calculated.

### CEM‐Cas9 cell line

Lentiviral packaging utilized the lentiCas9 plasmid (Addgene plasmid #52962). CEM cells were exposed to lentiviral particles, incubated for 48 h, and selected with blasticidin for 7 days. Single‐cell sorting (BD FACS Aria III) was performed. CEM‐Cas9 cell lines were expanded, and knock‐in was confirmed by western blot. Cas9 functionality was validated by introducing a CD95‐targeting sgRNA and quantifying CD95 surface expression via flow cytometry (Table S1).

### Genome‐wide CRISPR/Cas9 knockout library screening

The Human Brunello CRISPR knockout library (Addgene #73178), with 76 441 sgRNA sequences targeting 19 114 genes, was used for screening. sgRNA sequences were included in the lentiGuide‐Puro plasmid for lentiviral packaging. To achieve a 400‐fold library coverage, a minimum of 9 × 10^7^ cells per sample was used. CEM‐Cas9 cells were transduced with the sgRNA library (low MOI, ~0.3), followed by a 48‐h incubation and a 7‐day puromycin selection. Post‐selection, samples were expanded to a minimum of 6 × 10^7^ cells. For each sample, 3 × 10^7^ cells were harvested as a day 0 control. Two samples were treated with either DMSO (vehicle control) or 18 μm DMF for approximately 10 cell cycles (12 days). Genomic DNA was extracted, sgRNAs were PCR‐amplified, and products were sequenced on an Illumina NextSeq 550.

### Establishment of EZH2 knockout cell lines and eGFP control cell lines

The Cas9 lentiCas9‐Puro backbone facilitated lentiviral packaging of sgRNAs targeting EZH2, designed using Geneious Prime Software. Lentiviruses with two different sgRNAs were transduced at a low MOI (~0.3) onto CEM cells, followed by puromycin selection for 7 days. Mutant cell pools, validated by western blot (EZH2 antibody), were expanded. For stable eGFP overexpression, CEM cells were transduced with eGFP‐carrying lentiviruses, and single‐cell sorting ensured pool homogeneity. Supplementary information includes sgRNA sequences (Table S1).

### Flow cytometric analysis with patient‐derived CD4 ^+^ T cells

Primary CD4+ T cells from Sézary syndrome patients were seeded at 500000 cells/ml in 48‐well plates. After 24 h, cells were treated with DMF alone or with the respective EZH2/PC2 inhibitors at indicated concentrations for an additional 48 h at 37 °C and 5% CO_2_. For flow cytometric analysis, T cells were incubated in 500 μL with BD Pharmingen™ FITC Annexin V (1 : 100 dilution) and BD Pharmingen™ DAPI Solution (1 : 10 000 dilution) in PBS. Measurements were conducted by flow cytometer.

### EZH2 knockout cell competition assay

CEM transduced with an empty sgRNA backbone, CEM‐EZH2‐KO‐sgRNA#3 and CEM‐EZH2‐KO‐sgRNA#5 cells were cocultured 1 : 1 with CEM‐eGFP cells. Three samples were treated with DMSO (ctrl.) or 18 μm DMF, incubated at 37 °C and 5% CO_2_. Cell suspensions were collected at day 0, 2, 4, 8, and 11, and eGFP‐positive cell proportions were determined using an LSRFortessa (BD) flow cytometer with FACSDiva™ software (BD). Specific changes in the eGFP+ proportion were calculated over time.

*Specific change from baseline* (time‐dependent) was calculated using the following equation:

$$\text{Specific change from baseline}\;\left[\%\right]=\frac{\%\text{eGFP}{+}_{\text{dayX}}-\%\text{eGFP}{+}_{\mathrm{day}0}\;}{100-\%\text{eGFP}{+}_{\mathrm{day}0}}$$

*Specific change from baseline* (treatment‐dependent) was calculated using the following equation:

$$\text{Specific change from baseline}\;\left[\%\right]\;\left(\text{treatment dependent}\right)=\frac{\%\text{specific ΔeGFP}{+}_{\text{treated}}-\%\text{specific ΔeGFP}{+}_{\text{control}}\;}{100-\%\text{specific}\;\Delta \text{eGFP}{+}_{\text{control}}}$$

### Cell death assay

Cell death was analyzed by FSC/SSC or Annexin V‐FiTC/DAPI staining using flow cytometry as described previously [77].

*Specific cell death* was calculated using the following equation [78].

$$\text{Specific cell death}\;\left[\%\right]=\frac{\%{\text{Cell death}}_{\text{treated}}\;\left(Q2+Q3\right)-\%{\text{Cell death}}_{\text{control}}\;\left(Q2+Q3\right)}{100-\%{\text{Cell death}}_{\text{control}}\;\left(Q2+Q3\right))}$$

### Western blot analysis

Western blotting was conducted as described previously [79]. For histone extraction, cell lysates were prepared with the Histone Extraction Kit (abcam, ab113476). Primary and secondary antibodies included anti‐CRISPR/Cas9 (1:1000, NBP2‐36440, novusbio), anti‐Di‐Methyl‐Histone H3 (1:1000, #9728, Cell Signaling, Leiden, the Netherlands), anti‐Tri‐Methyl‐Histone H3 (1:1000; #9733, Cell Signaling), anti‐Histone H3 (1:1000, 9715, Cell Signaling), anti‐beta actin HRP‐conjugated (1:10 000, ab49900, Abcam, Amsterdam, the Netherlands), anti‐mouse IgG HRP‐conjugated (1:2500, ab6728, Abcam), and anti‐rabbit IgG HRP‐conjugated (1:20 000, 31 460, Thermo Fisher Scientific). Densitometric analysis was conducted using imagej.

### Bioinformatic analyses

Bioinformatic analysis containing NGS quality control, sgRNA read count, and hit calling was conducted following the MAGeCKFlute pipeline [80]. Screen hits were identified using the provided FASTQ sequencing data processed with the MAGeCK MLE algorithm on a Python 3.7 system. Quality control, modeling, and visualization have been performed through the MAGeCK‐Vispr workflow [80].

### Software

Graphical plots and statistics were analyzed using GraphPad Prism. Graphical figures were created using Inkscape and Biorender.com. Densitometric analyses of immunoblots were performed with imagej, while the processing of immunoblots and agarose gels utilized ImageLab software. Plasmids were designed and visualized with Geneious Prime. Flow cytometry data were analyzed using FACSDiva™ (BD) and FlowJo (BD). Bioinformatic analyses were conducted using Python 3.7 and R platforms running on MacOS.

## Conflict of interest

The authors declare no conflict of interest.

## Author contributions

KG initiated the project; KG and DT designed the work; KG, DT, JPT were responsible for experimental design; JPT, DT, JPN, TH, KP, MG, BV, and EA performed the experiments; JPT, DT, KG, JPN, and MM analyzed the data and contributed to the interpretation of the results; JPN, TH, AK, PH, PLB, LT, and PM collected and processed human samples and were involved in experimental evaluation; EA, DT, FV, MK, and TH isolated T cells and supported data analysis; JPT and DT drafted the manuscript and prepared figures; JPT, DT, and KG wrote the manuscript; MM, CK, and KG drafted and critically revised the manuscript. All authors approved the final version of the manuscript.

## Supporting information


**Fig. S1.** Lentiviral transduction and generation of functional Cas9 and CD95 knockout in CEM cells.
**Fig. S2.** Lentiviral sgRNA constructs and competition assays in CEM cells.
**Fig. S3.** Tazemetostat reduces H3K27 di‐ and tri‐methylation in CEM and HH cells after 96 h.
**Fig. S4.** EZH2 inhibition by A‐395 and MAK638 decreases H3K27 methylation in CEM and HH cells after 96 h.
**Table S1.** Overview of sgRNA sequences utilized in this study.

## Data Availability

The sequencing data generated in this study have been deposited in the Sequence Read Archive (SRA) database and are accessible under the assigned accession number PRJNA1160780.
