# Supplementary material for: CRISPR‐Cas9 screen reveals that inhibition of enhancer of zeste homolog 2 sensitizes malignant T cells to dimethyl‐fumarate‐induced cell death
Source: FEBS J. 2025 Aug 1;293(3):749–65. doi: 10.1111/febs.70208 (PMC12871913; doi:10.1111/febs.70208)
Supplement: Supplementary file 1 — Fig. S1. Lentiviral transduction and generation of functional Cas9 and CD95 knockout in CEM cells. Fig. S2. Lentiviral sgRNA constructs and competition assays in CEM cells. Fig. S3. Tazemetostat reduces H3K27 di‐ and tri‐methylation in CEM and HH cells after 96 h. Fig. S4. EZH2 inhibition by A‐395 and MAK638 decreases H3K27 methylation in CEM and HH cells after 96 h. Table S1. Overview of sgRNA sequences utilized in this study. [file FEBS-293-749-s001.docx]

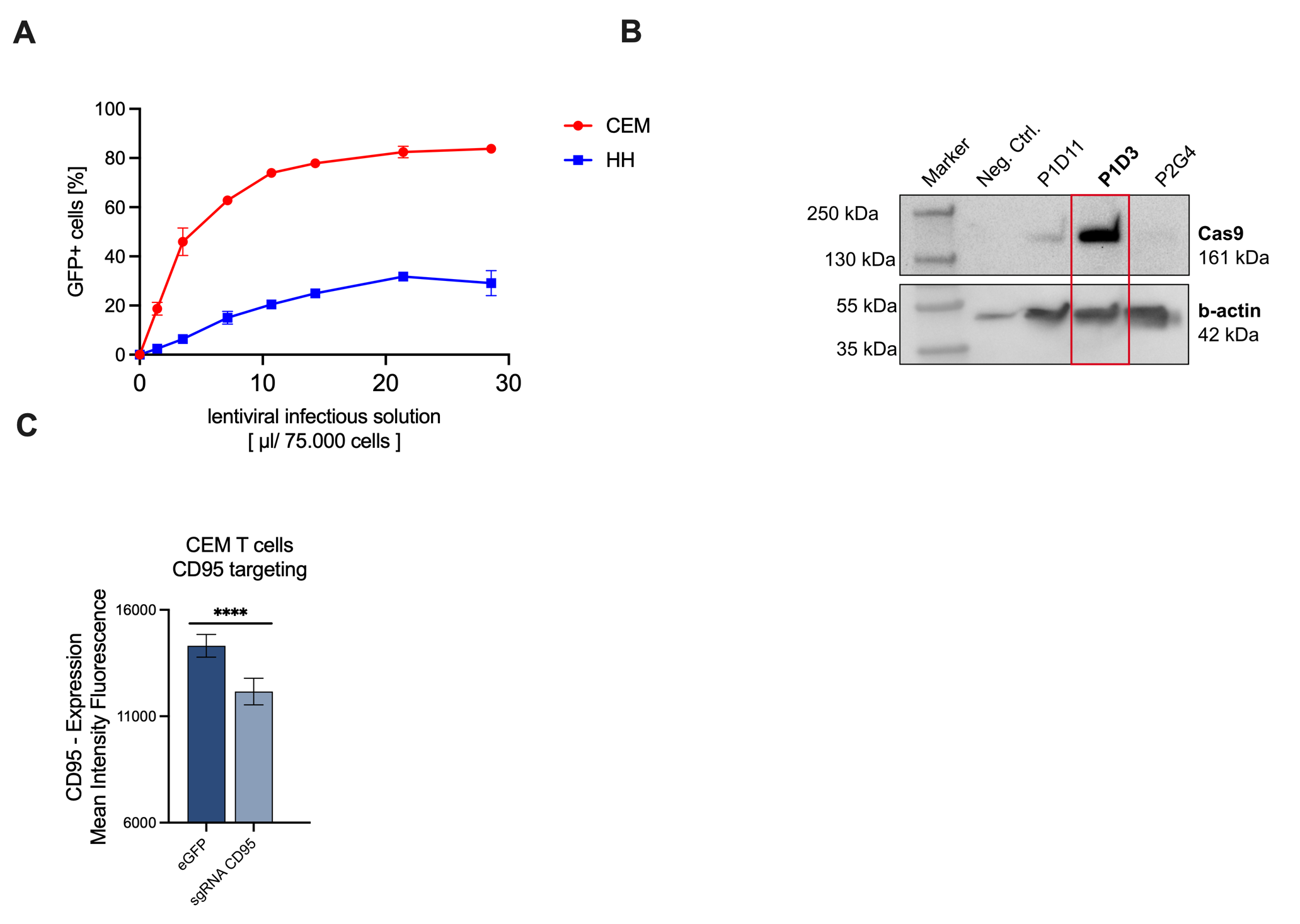


**Supplementary Figure 1: Lentiviral transduction and generation of functional Cas9 and CD95 knockout in CEM cells. A** Analysis of transduction efficacy in CEM and HH cells. Cells were treated with increasing volumes of an infectious solution containing lentiviral particles carrying GFP. The percentage of GFP-positive cells was determined 48 hours post-transduction. **B** Western blot analysis of different CEM-Cas9 clones after selection and sorting. Clone P1D3 showed a robust Cas9 overexpression and was selected for the subsequent experiments. **C** CD95 quantification of CEM-CD95-KO cells showed a significantly lower CD95 expression on CD95-KO cells compared to eGFP-transduced vehicles. n = 12 biologically independent samples. Source data are provided as a Source

Data file. Student’s t-test ****P < 0.0001.


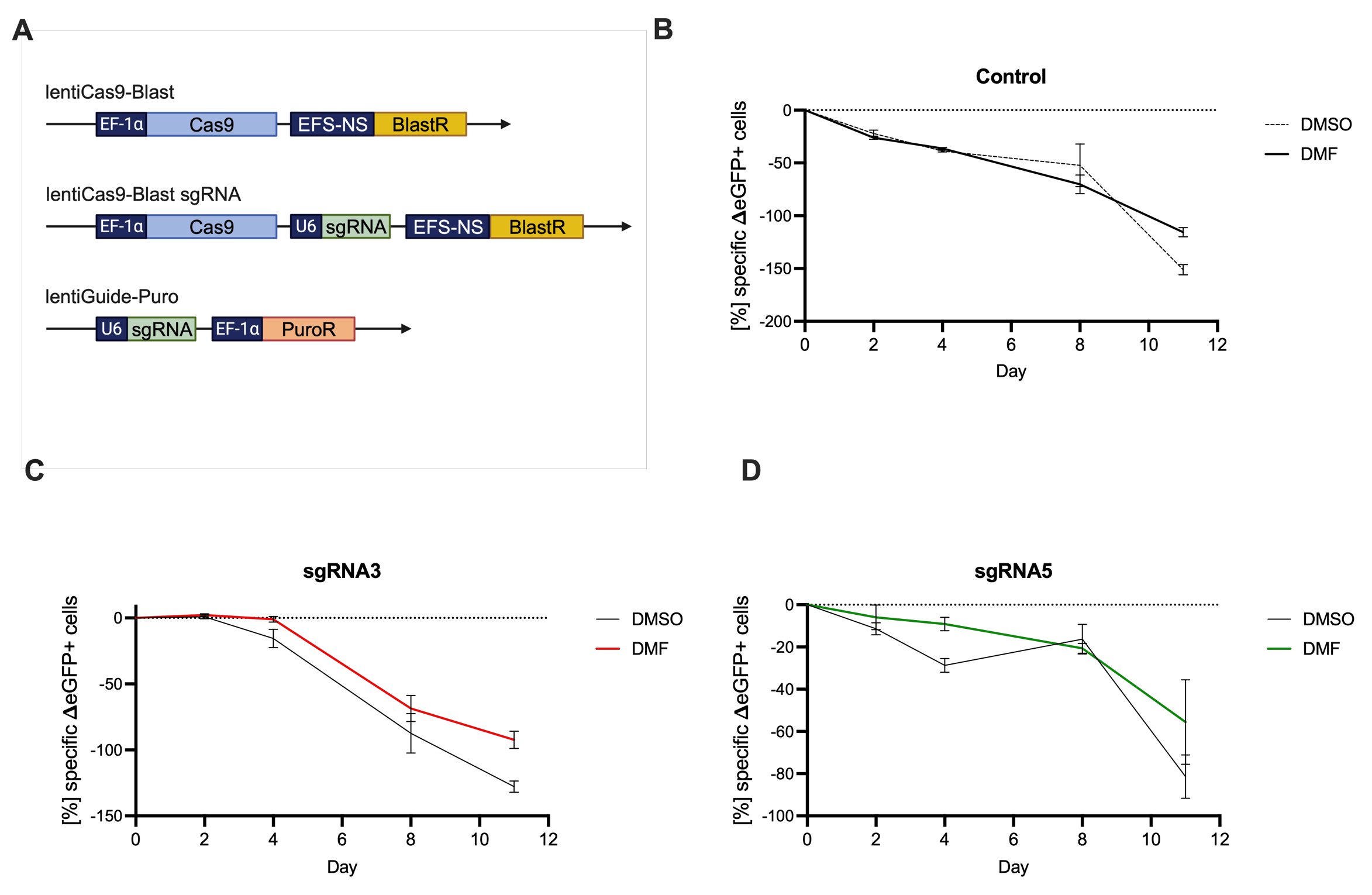


**Supplementary Figure 2: Lentiviral sgRNA constructs and competition assays in CEM cells. A** Overview of used lentiviral plasmids. LentiCas9-Blast was modified by cloning. A U6 promotor and the sgRNA sequences of CD95 and EZH2 targeting were added. sgRNA sequences are listed in the Supplementary Table S1. Figure was created using biorender.com.  **B** Competition assay of CEM cells with an empty sgRNA backbone mixed at a 1:1 ratio with CEM-GFP+ cells. Cells were treated with either DMSO or 18µM DMF. The proportion of CEM-GFP+ cells were measured on day 0, 2, 4, 8, and 11. Specific changes have been calculated and displayed. **C** Competition assay of CEM-sgRNA3 mixed at a 1:1 ratio with CEM-GFP+ cells. Cells were treated with either DMSO or 18µM DMF. The proportion of CEM-GFP+ cells were measured on day 0, 2, 4, 8, and 11. Specific changes have been calculated and displayed. **D** Competition assay of CEM-sgRNA5 mixed at a 1:1 ratio with CEM-GFP+ cells. Cells were treated with either DMSO or 18µM DMF. The proportion of CEM-GFP+ cells were measured on day 0, 2, 4, 8, and 11. Specific changes have been calculated and displayed. Error bars in panel B-D represents the standard deviation (SD). Experiments were conducted in triplicate (n=3) at each time point.


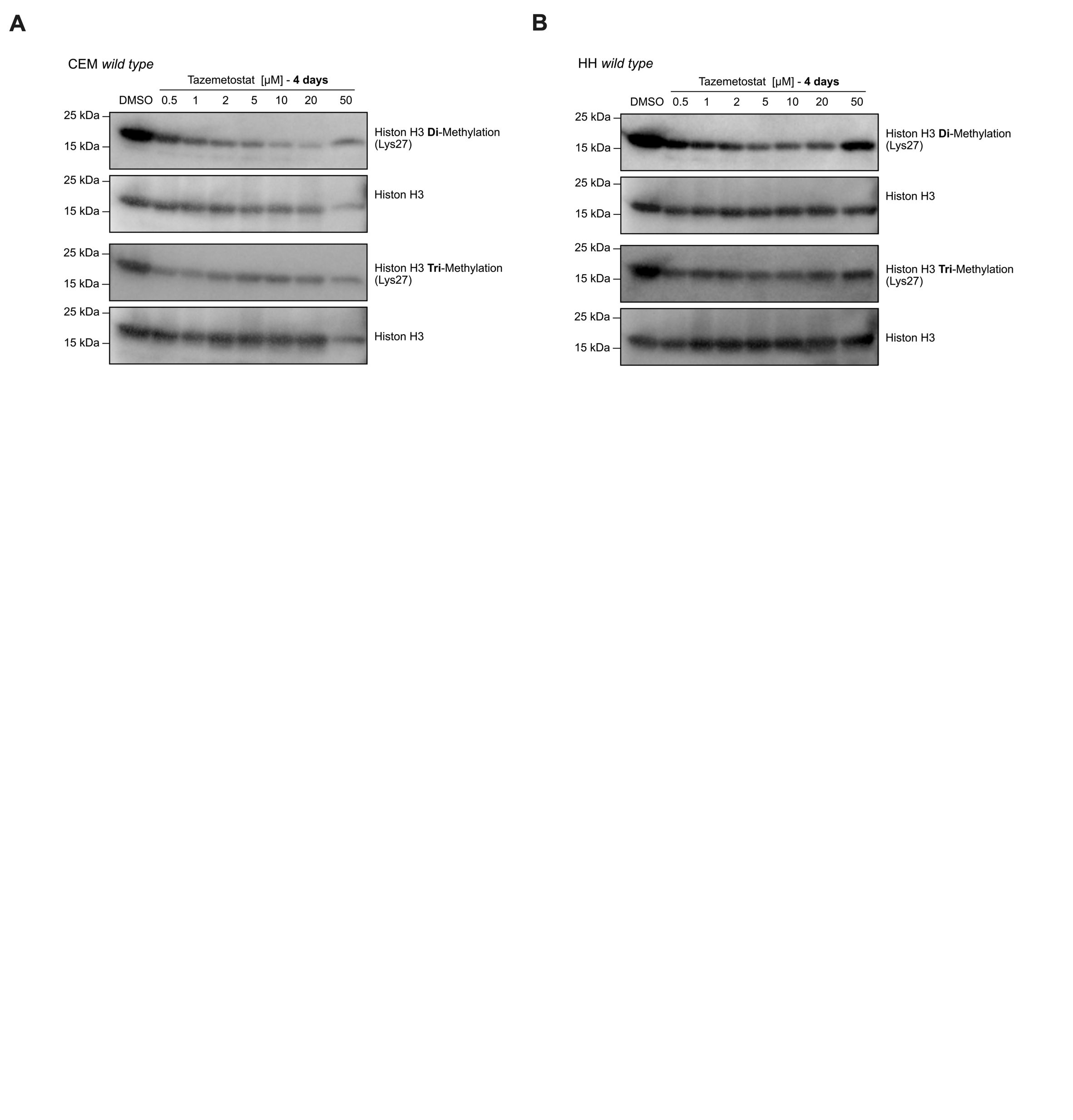


**Supplementary Figure 3: Tazemetostat reduces H3K27 di- and tri-methylation in CEM and HH cells after 96h.** Western blot analysis of di- and tri-methylation of Lysin 27 on histone H3 (H3K27) in CEM **A** and HH **B** cells treated with increasing concentrations of tazemetostat for 96h. The upper panel shows the amount of di- or tri-methylated H3K27. The lower panel shows the amount of Histon 3, used as loading control. n = 3 biologically independent samples in both panels.


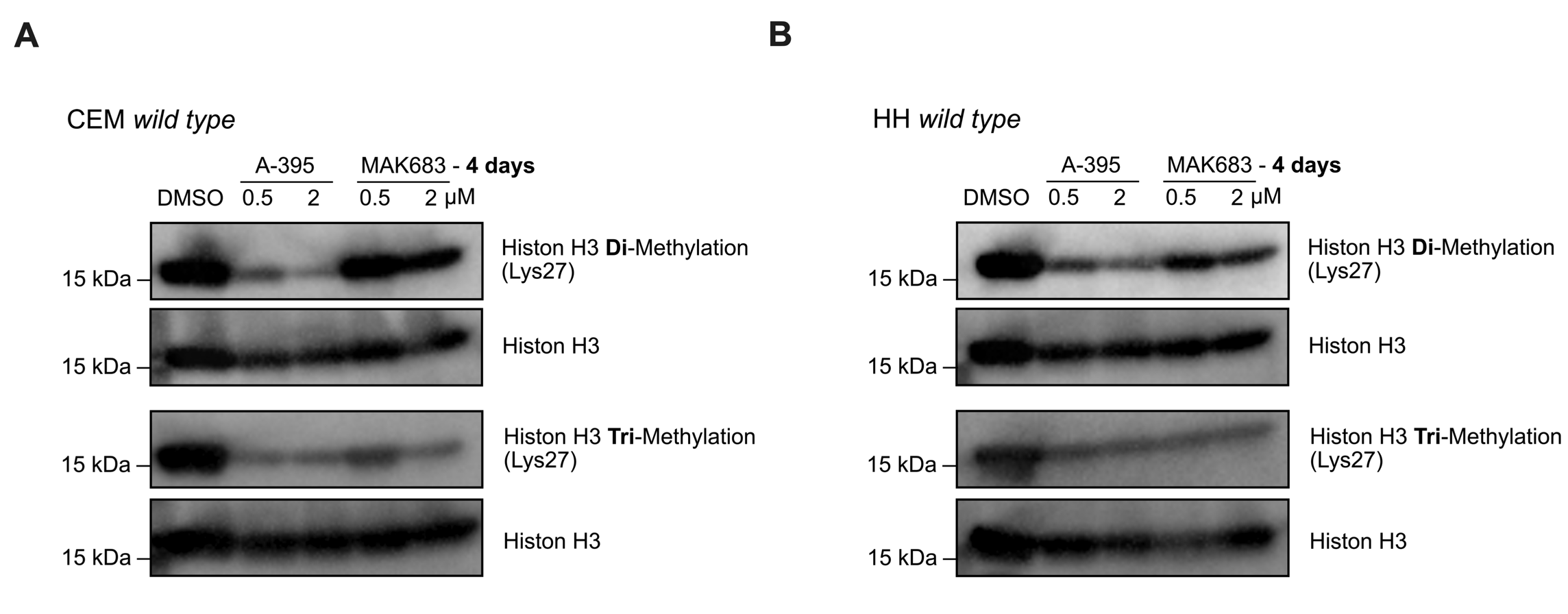


**Supplementary Figure 4: EZH2 inhibition by A-395 and MAK638 decreases H3K27 methylation in CEM and HH cells after 96h.** Western blot analysis of di- and tri-methylation of Lysin 27 on histone H3 (H3K27) in CEM **A** and HH **B** cells treated with different concentrations of A-395 (0.5/2µM) and MAK638 (0.5/2µM) for 96h. The upper panel shows the amount of di- or tri-methylated H3K27. The lower panel shows the amount of Histon 3, used as loading control. n = 3 biologically independent samples in both panels.

| **Supplementary Table S1** | |
| --- | --- |
| **sgRNA** | **Sequence** |
| **sgRNA CD95#3** | GGAGTTGATGTCAGTCACTTTGCTGGAAACAGCATAGCAAGTTTAAATAAGGCTAGTCCGTTATCAACTTGAAAAAGTGGCACCGAGTCGGTGCT |
| **sgRNA EZH2#3** | GCCCTTATCTGGAAACATTGAGGTGCTGGAAACAGCATAGCAAGTTTAAATAAGGCTAGTCCGTTATCAACTTGAAAAAGTGGCACCGAGTCGGTGCT |
| **sgRNA EZH2#5** | TTATGATGGGAAAGTACACGTGCTGGAAACAGCATAGCAAGTTTAAATAAGGCTAGTCCGTTATCAACTTGAAAAAGTGGCACCGAGTCGGTGCT |

**Supplementary Table S1:** Compilation of single guide RNAs (sgRNAs) used.
